# Antimicrobial, Shelf-Life Stability, and Effect of Maltodextrin and Gum Arabic on the Encapsulation Efficiency of Sugarcane Bagasse Bioactive Compounds

**DOI:** 10.3390/foods10010116

**Published:** 2021-01-08

**Authors:** Victor Velazquez-Martinez, Delia Valles-Rosales, Laura Rodriguez-Uribe, Omar Holguin, Julian Quintero-Quiroz, Damian Reyes-Jaquez, Manuel Ivan Rodriguez-Borbon, Luz Yazmin Villagrán-Villegas, Efren Delgado

**Affiliations:** 1 Industrial Engineering, New Mexico State University, Las Cruces, NM 88003, USA; yambe@nmsu.edu (V.V.-M.); dvalles@nmsu.edu (D.V.-R.); ivan.rodriguez@uacj.mx (M.I.R.-B.); 2 Department of Family and Consumer Sciences, Food Science and Technology, New Mexico State University, Las Cruces, NM 88003, USA; 3 Plant and Environmental Sciences, New Mexico State University, Las Cruces, NM 88003, USA; laurodri@nmsu.edu (L.R.-U.); frholgui@nmsu.edu (O.H.); 4 Facultad de Ciencias Farmacéuticas y Alimentarias, Universidad de Antioquia, University Campus, Medellin 050010, Colombia; julian.quintero@udea.edu.co; 5 Posgrado en Ingenieria Quimica, Instituto Tecnologico de Durango Durango, Durango 34080, DGO., Mexico; damian.reyes@itdurango.edu.mx; 6 Facultad de Ingenieria Mecanica Electrica, Universidad Veracruzana, Poza Rica 93390, VER., Mexico; yvillagran@uv.mx

**Keywords:** polyphenol compounds, microencapsulation, antimicrobial activity, antioxidant activity, thermal stability, shelf-life stability

## Abstract

This study shows the effects of maltodextrins and gum arabic as microencapsulation agents on the stability of sugarcane bagasse extracts and the potential use of the extracts as antimicrobial agents. The bioactive compounds in sugarcane bagasse (SCB) were extracted using 90% methanol and an orbital shaker at a fixed temperature of 50 °C, thereby obtaining a yield of the total phenolic content of 5.91 mg GAE/g. The bioactive compounds identified in the by-product were flavonoids, alkaloids, and lignan (-) Podophyllotoxin. The total phenolic content (TPC), antioxidant activity, and shelf-life stability of fresh and microencapsulated TPC were analyzed. This experiment’s optimal microencapsulation can be obtained with a ratio of 0.6% maltodextrin (MD)/9.423% gum arabic (GA). Sugarcane bagasse showed high antioxidant activities, which remained stable after 30 days of storage and antimicrobial properties against *E. coli*, *B. cereus*, *S. aureus*, and the modified yeast *SGS1*. The TPC of the microencapsulated SCB extracts was not affected (*p* > 0.05) by time or storage temperature due to the combination of MD and GA as encapsulating agents. The antioxidant and antimicrobial capacities of sugarcane bagasse extracts showed their potential use as a source of bioactive compounds for further use as a food additive or nutraceutical. The results are a first step in encapsulating phenolic compounds from SCB as a promising source of antioxidant agents and ultimately a novel resource for functional foods.

## 1. Introduction

Agro-industrial by-products have recently experienced an increased application as a source of bioactive compounds being used as ingredients in food, animal feeds, and aquacultural feeds [1]. Agro-industrial by-products such as orange bagasse, glandless cottonseed meal, apple pomace, and broken beans, among others, can be used as food and feed additives. The bioactive compounds in agro-industrial by-products such as proteins, fibers, and phenolic compounds can be extracted and used as additives or substitutes in food products [2,3,4].

Sugarcane (*Saccharum officinarum*), as a grass, has its origins in New Guinea and is mostly found in tropical and semitropical regions worldwide. Brazil is the highest contributor to sugar’s world production, followed by China, India, Mexico, and, recently, the United States [5]. Research has shown that the bioactive compounds present in the sugarcane can be beneficial for health. For example, policosanol (long-chain fatty alcohols) extracted from sugarcane wax can lower the values of total cholesterol and LDL-cholesterol in the blood while improving HDL-cholesterol [6]. The phenolic compounds present in the sugarcane juice reduce weight loss in rats, particularly those intoxicated with methylmercury chloride [7]. The sugarcane industry produces four main by-products, described as sugarcane tops, bagasse, filter muds, and molasses. The total worldwide production of sugarcane was over 186 million metric tons in 2018, and bagasse accounted for 28% of the said annual production [8]. Thus, about 52 million tons of sugarcane bagasse is produced every year worldwide, which constitutes a potential source of these compounds for human health benefit. Sugarcane bagasse is used mostly as biomass, animal feed for ruminants, molded products used for kitchen furniture, chair backs, seats, and alcohol production. However, sugarcane bagasse still contains flavonoids, phenolic acids, and other phenolic compounds with antioxidant activity [5,9]. Among the most common phenolic compounds found in sugarcane bagasse are genistin, p-coumaric acid, quercetin, and genistein [10,11]. These bioactive compounds have been reported to exhibit anti-adipogenesis activity and therapeutic properties. The bioactive compounds quercetin, genistin, genistein, and coumaric acid present in sugarcane showed immunomodulatory activity and can also be used as a potential antidiabetic agent [11,12,13].

Only limited research has focused on identifying bioactive compounds present in sugarcane bagasse and their antioxidant and antimicrobial activities. Different extraction methods and solvents have been used for this purpose, such as sonication with ethanol, and separation with ethyl acetate, n-butanol, and petroleum ether [11] or by pyrolysis [14]. The bioactive compounds identified in different studies are different due to their solvent and concentration, and the polarity affinity of each bioactive compound [11]. The antioxidant and antimicrobial activity exhibited by the extracted phenolic compounds can be compromised or degraded due to several factors such as pH, light exposure, or temperature. The microencapsulation of bioactive compounds offers protection from the factors mentioned above and maintains its stability while increasing its shelf life with gamma encapsulation agents’ help. Some microencapsulation techniques are freeze drying, spray drying, coacervation, and emulsions [15].

The antioxidant capacity, shelf-life stability, and antimicrobial activity of bioactive compounds extracted from sugarcane bagasse have not been extensively studied. However, there is a handful of research findings related to the encapsulation of bioactive compounds from different plants [16,17] and natural by-products [18,19] but there is no literature about the encapsulation of bioactive compounds extracted from sugarcane bagasse (SCB). This research aims to analyze microencapsulation’s effect on the stability of sugarcane bagasse extracts and their potential use as antioxidant and antimicrobial agents.

## 2. Materials and Methods

The SCB was retrieved randomly from the “Mahuixtlan” sugar mill located in Veracruz, Mexico. The SCB was dried at 55 °C in a Blue M oven (Lindberg/MPH, Riverside, MI, USA) until 0.9% moisture content was obtained and milled to a particle size < 500 µm using a Wiley mill Model 4 (Thomas Scientific, Swedesboro, NJ, USA) (Table 1). The moisture content was measured using a moisture analyzer IR60 (Denver Instrument, Bohemia, NY, USA). The bagasse samples were stored in vacuum-packed bags at −20 °C for further use.

### 2.1. Chemicals

Gallic acid and Phenazine Methosulfate (PMS) were purchased from ACROS Organics (ACROS Organics, Geel, Belgium). Folin–Ciocalteu and p-Iodonitrotetrazolium Violet (INT) were purchased from MP Biomedicals (MP Biomedicals, LLC, Irvine, CA, USA). 2,2-diphenyl-1-picryl-hydrazyl (DPPH) and the antibiotic Ampicillin were purchased from Alfa Aesar (Alfa Aesar, Tewksbury, MA, USA). Tris-HCl was purchased from Promega (Promega Co., Madison, WI, USA). β-Nicotinamide adenine dinucleotide (NADH), 2,4,6-tripyridyl-s-triazine (TPTZ), Nitro Blue Tetrazolium (NBT), and HPLC grade Methanol were purchased from MilliporeSigma (MilliporeSigma, Burlington, MA, USA).

### 2.2. Sugarcane Bagasse Characterization

The nutrient composition of three samples of SCB was assessed using the methods prescribed by the Association of Official Agricultural Chemists (AOAC 2005). The moisture content (AOAC 925.45), total mineral content (Ash) (AOAC 942.05), crude fat ether extraction (AOAC 2003.05); crude protein (CP) content were measured using the Kjeldahl method N × 6.25 (AOAC. 991.20) and Ca, P, Mg, K, Na, Fe, Zn, Cu, Mn, Mo, and S were determined by microwave digestion of samples and inductively coupled plasma-atomic emission spectrometry (AOAS 2011.14). The acid detergent fiber (ADF) and neutral detergent fiber (aNDF) were determined as previously described [2]. Non-fiber carbohydrates (*NFC*) were calculated as follows [2]:

(1) NFC=100−((% NDF−% NDF−CP)+% CP+% Fat+Ash)

where *NDF* = Non-fiber carbohydrates, *CP* = crude protein.

### 2.3. SCB Extracts

The extraction of bioactive compounds was done using 1 g of SCB with 10 mL of 90% methanol. The combination was mixed in an orbital shaker MaxQ HP Incubated Tabletop Orbital Shaker (ThermoFisher Scientific, Waltham, MA, USA) at 120 rpm for 48 h at 50 °C. After extraction, the supernatant was collected by centrifugation at 3000 *g* for 10 min and filtered using grade 1 Whatman filter papers (Whatman plc Co., Maidstone, United Kingdom) and stored at 4 °C.

### 2.4. SCB Lyophilized Powder

SCB extracts were concentrated by rotoevaporation (Heidolph Laborota, 4000 efficient, Schwabach, Germany), frozen with liquid nitrogen, and then dried by lyophilization (FreeZone Labconco, Kansas City, MO, USA). The lyophilized powder was stored at 4 °C for further use.

### 2.5. Total Phenolic Content

The Total Phenolic Content (TPC) of the extract was measured using the Folin–Ciocalteu method [20]; however, with some modifications, 0.5 mL of Folin–Ciocalteu was added to 100 µL of the sample and 900 µL of distilled water. After the mixture was kept in the dark for 5 min, 1.5 mL of 20% Na_2_CO_3_ and distilled water was added to obtain a final volume of 10 mL and mixed using a vortex mixer. The solution was incubated at 75 °C for 10 min, and the absorbance was read at 760 nm in a Genesys 10S UV-Vis spectrophotometer (ThermoFisher Scientific, Waltham, MA, USA). A standard calibration curve was prepared using gallic acid with concentrations ranging from 0.09 to 1 mg/mL. TPC is presented as gallic acid equivalents per gram of dry matter of SCB (mg/GAE). The TPC values were used as the response variable to conduct statistical analysis.

### 2.6. DPPH Scavenging Activity

The antioxidant activity (AA) was measured by the DPPH method [20] with minor modifications. 100 µL of the extract and 3 mL of a methanolic solution of DPPH (0.1 mM) were vortexed and kept in the dark for 15 min at room temperature. The absorbance was measured at 517 nm using a spectrophotometer against a methanol blank. The *inhibition* of free *radicals* was calculated using:

(2) $$\%\;inhibition\;of\;DPPH\;radical=\left(\left[A_{0}-A_{s}\right]/A_{0}\right)x100$$

where *A*_0_ is the absorbance of the control (3 mL of *DPPH* reagent + 100 µL of methanol), the same as the absorbance of the sample.

### 2.7. Ferric Reducing Antioxidant Power (FRAP)

The FRAP method [20] was used with slight modifications. The FRAP reagent for the sample was prepared by mixing acetate buffer (300 mM, pH 3.6), TPTZ (10 mM) dissolved in HCl (40 mM), and FeCl_3_·6H_2_O (20 mM) dissolved in DI water, in a ratio of 10:1:1, respectively. FRAP reagent for calibration curve was prepared in the same manner, but we used DI water instead of FeCl_3_·6H_2_O (20 mM). FeSO_4_·7H_2_O (0.001 M) was used as a standard solution. 2 mL of FRAP reagent was added to a mixture of 100 μL of the sample (or standard solution) and 900 μL of distilled water. After mixing vigorously, all tubes were incubated at room temperature in the dark for 30 min. The absorbance (593 nm) was measured against the blank (1 mL water mixed with 2 mL FRAP reagent for calibration curve). FRAP values were expressed as µmoles of ferrous equivalent Fe (II).

### 2.8. Superoxide Radical Scavenging (SOD) Method

The measurement of Superoxide Radical Scavenging activity is based on the generation of anion radicals within the PMS/NADH system and the reduction of NBT using the method described by [20]. The superoxide anion radicals were generated in 3.0 mL of Tris–HCl buffer (16 mM, pH 8.0), containing 0.5 mL of NBT (0.3 mM), 0.5 mL NADH (0.936 mM) solution, 1.0 mL extract and 0.5 mL Tris–HCl buffer (16 mM, pH 8.0). 0.5 mL of 0.12 M PMS solution was added and incubated at 25 °C for 5 min. The absorbance was measured at 560 nm against the blank sample. The inhibition of free radicals was calculated using Equation (2) (Shown above), where *A*_0_ is the absorbance of the blank reagent 2, which is the same as the sample’s absorbance.

### 2.9. Liquid Chromatography-Mass Spectrometry

Lyophilized powder extracts were suspended in methanol HPLC grade in a ratio of 2 mg/mL for HPLC-MS analysis [11]. Mass spectrometry was performed on a quadrupole time-of-flight (QTOF) mass spectrometer (QTOF Ultima, Waters, Manchester, UK). The spectrometer was equipped with a Lockspray™ electrospray ion source coupled to a Waters Acquity UPLC system (Waters, Manchester, UK). Mass spectra were collected in negative electrospray ionization mode (ESI-). The nebulization gas was set to 650 L/h at a temperature of 500 °C. The cone gas was set to 15 L/h, and the source temperature was set to 110 °C. Capillary voltage and cone voltage were set to 2500 V and 35 V, respectively. The microchannel plate detector voltage was set to 2200 V. The Q-TOF Ultima MS acquisition rate was set to 0.25 s with a 0.1 s interscan delay. The scan was set at a range of 100 to 1500 *m*/*z* to collect data continuously. A 50 ppm raffinose (503.1612 *m*/*z*) lock mass solution in 50:50 water; methanol was delivered at 20 μL/min through an auxiliary pump and collected every 10 s during the MS acquisition. A Waters BEH C18 column (2.1 × 50 mm, particle size 1.8 μm) was used. The Waters system was equipped with an ACQUITY Binary Solvent Manager and ACQUITY Sample Manager (20 μL sample loop, partial loop injection mode, 5 μL (Cell extracts) or 10 μL (Supernatant) injection volume, 4 °C. Eluents A and B were water and acetonitrile, respectively, both containing 0.1% formic acid. Elution was performed isocratically for 0.1 min at 8% eluent B, then a linear gradient with 100% eluent B in 6.0 min, and subsequently for 1 min at 100% eluent B. The column was equilibrated for 1.5 min at a flow rate of 500 μL/min at 40 °C. This equipment offered the advantage of running LC-MS/MS analysis.

Five different runs were used to conduct an alignment analysis in Ms-dial software with an Ms1/Ms2 tolerance of 0.1 Da and 0.5 of sigma value as a deconvolution parameter for identifying compounds. Moreover, a least-square regression model is used by MS-dial on unique ions to analyze and compare the spectra of the metabolites in the sample. In this manner, the compound’s identification is vastly improved [21]. The report of identified compounds was based on a reference match with the MSMS public negative V14 library [22]. For each identified biocompound there was a description of the m/z similarity, mass (Da), ion abundance or absolute abundance (AA), and Relative Standard Deviation (RSD) percentage.

AA was given as a number of ions in the mass spectrometer, and it was determined by MS dial software by features in the vicinity of the (t, *m*/*z*) location of those species identified by MS/MS satisfying a specified quality threshold. Absolute abundance was calculated by MS dial software by the peak height/area ratio. The species’ spectral count is the number of MS/MS identifications of that species satisfying the threshold. The species’ ion abundance is determined by fitting the corresponding feature to the model f ≡ f(*t*, *m*) which is described by other authors [23].

For *RSD* percentage, the following Dolan’s rule of thumb was used:

(3) $$RSD\;\left(\%\right)=\;\frac{50}{\left(\frac{s}{n}\;ratio\right)}$$

*s*/*n* is the signal to noise ratio obtained from Ms-dial results. An upper limit of 10% was used as a cut off for this bioanalytical project [24].

### 2.10. Antimicrobial Activity from Sugarcane Bagasse Extracts

The antimicrobial activity of SCB extracts was tested on three different bacteria (*E. coli K12* [ATCC^®^ 8739], *B. cereus* [ATCC^®^ 11778], and *S. aureus* [ATCC^®^ 6538]) and one modified yeast (Slow Growth Suppressor 1 or *SGS1* [ATCC^®^ 4010775]) [15]. Additionally, the antibiotic Ampicillin (AMP) was used for comparison with SCB extracts. Briefly, 50 mg of lyophilized powder from SCB extracts (or AMP) was accurately weighed and suspended in 5 mL of DMSO: MeOH in a ratio of 1:1 (DMSO: DI water for AMP). A series of dilutions from the stock solution ranging from 0.8 to 800 mg/mL were prepared and filtered through a sterile syringe filter (0.25 µm). From each dilution, 20 µL was deposited in microplate wells along with 220 µL of the media used to grow each microorganism. 10 µL from each microorganism suspended in media (measured at 0.1 of absorbance at 630 nm) was added to each well. The covered microplate was incubated overnight at a particular temperature to grow each microorganism. After incubation, 40 µL of 0.02 mg/mL INT was added to the microplate and further incubated for one hour. The color change was measured in a microplate reader (ELx808, Biotek Instruments Inc., Winooski, VT, USA) at 630 nm, where the colorless INT changed to red with the growth of microorganism. The growth *inhibition* was calculated using Equation (4):

(4) $$Inhibition\;\left(\%\right)=\left(100-\left(A_{s}*100\right)\right)/\left(A_{t1}+\;A_{t2}\right)$$

where *A_s_* is the absorbance of the sample, *A_t_*_1_ is the absorbance of the sample without microorganism, and *A_t_*_2_ the absorbance of the microorganism without treatment. The minimum inhibitory concentration is the first well concentration that did not show the color change (from yellow to red).

### 2.11. Microencapsulation of Bioactive Compounds

SCB extracts were concentrated by rotoevaporation, and the resulting extract had 7.1 °Brix. Maltodextrin (MD) and gum arabic (GA) were used as micro-encapsulating agents. The core to coating ratio was 1:5, with a total of 10% (*w*/*w*) of encapsulating agents. The coating solutions were prepared using a homogenizer POLYTRON Ch-6010 PT 10-35 (Kinematica, Bohemia, NY, USA) at 7000 rpm for 1 min and stored in the refrigerator overnight for hydration. After hydration, each of the encapsulating agent solutions was mixed with 10 mL of the concentrated SCB extract and stored in an ultra-freezer. After freezing, all the solutions were freeze-dried for 48 hr. The response variables used in running the statistical analysis were encapsulation efficiency, DPPH, and FRAP.

### 2.12. Microencapsulation Efficiency

The TPC in microcapsules was measured as previously described [15]. First, 200 mg of microcapsules were mixed with 1 mL of acetonitrile and 1 mL of methanol; acetic acid; and water (50:8:42 *v*/*v*/*v*) to destroy the coat of the microcapsules. The surface phenolic content (SPC) was measured as follows: 200 mg were mixed with a solution of ethanol and methanol (1:1). Both TPC and SPC solutions were vortexed for 1 min and centrifuged at 3500 rpm for 5 min. The resulting supernatant was then filtered with 0.45 µm syringe filters. The phenolic content on both solutions was measured following the same Folin–Ciocalteu method as previously mentioned.

The encapsulation efficiency (*E.E.*) was calculated using Equation (5):

(5) $$E.E.\;\left(\%\right)=\left(\frac{TPC-SPC}{TPC}\right)*100$$

Accelerated shelf-life testing of free compounds and microcapsules at elevated temperatures

The thermal stability of micro-encapsulated compounds from SCB was determined at three different temperatures: 60, 80, and 100 °C. For the free compounds, 10 mg was used, and 200 mg for the micro-encapsulated samples. All vials were placed at the temperatures mentioned above, removing one vial every 2 h until the experiment was completed in 14 h. The reaction was stopped by placing the vial in an ice-water bath. The samples were kept in the ultra-freezer for further use. The results were calculated as relative (%) increase or decrease of TPC compared to the value at the start of the experiment [25]. The results will help to demonstrate the effectiveness of the protection of bioactive compounds by microencapsulation. The response variable to run statistics was TPC. For free compounds, the content of each vial was resuspended in 5 mL of methanol to get a final concentration of 2 mg/mL. For microencapsulated bioactive compounds, the same methodology to destroy the coat of the microcapsules was followed. The Folin–Ciocalteu method to determine TPC was used.

### 2.13. Statistical Analysis

A multi-paired t-test was performed using RStudio Statistical software (Version 1.2.1335, Boston, MA, USA) to determine the changes in antioxidant activity of fresh extracts and the antioxidant activity of the same extract after one month under storage conditions. A *p*-value of 0.05 was the cutoff for a significant difference. An experimental I-optimal design with a cubic model was used to obtain an optimal mixture between MD and GA to ensure the protection and stability of microencapsulated bioactive compounds and their antioxidant activities. Design-Expert version 11 (Stat-Ease Inc., Minneapolis, MN, USA) was used to obtain the experimental design.

## 3. Results and Discussion

### 3.1. Chemical Characterization of Sugarcane Bagasse

Table 1 shows the chemical characterization of sugarcane bagasse. The moisture content present in a forage needs to be lower than 15% to prevent mold growth [26]. The sugarcane bagasse (SCB) sample’s original moisture content was 45% and was lowered to 0.9% after oven-drying. SCB shows a low crude protein content (2.2%). Compared to other agro-industrial by-products, SCB has lower protein content than orange bagasse (6.9%) and glandless cottonseed meal (55%), but practically the same as that of apple pomace (2.2%) [2,4,27]. Because of the low protein content, SCB is not suitable for protein enrichment or substitute, but more appropriate for extracting the bioactive compounds.

SCB has a low-fat content (Table 1). The SCB found in orange bagasse, soybean, canola, and flaxseed meal has fat content well-below ideal concentration level [1,4]. The fat content in SCB could be used as an alternative to carnauba wax and fatty alcohol extraction. However, an in-depth analysis would be needed to find the correlation between the cost-benefit of the results.

The aNDF value of 82.6% (Table 1) is promising for industrial applications because it comprises the content of hemicellulose, cellulose, and lignin. Cellulose is used to obtain ethanol, while hemicellulose can be used to coat food items, control moisture on the surface, or for biomedical purposes. Lignin, if modified, can be used for animal feed and to produce nanostructured Langmuir–Blodgett films [28] with several applications in the fields of technology and biology. The aNDF and ADF values found in SCB are higher than the concentration levels found in apple pomace and orange bagasse [2,4]. SCB also has a low level of non-fiber carbohydrates, limiting its use as a source of starch in extruded products [29].

SCB has 4.5% ash content (Table 1), in which Fe and K are the main minerals present. The total mineral content is comparatively equal to the total mineral content in other agro-industrial by-products [1].

### 3.2. Total Phenolic Content

The total phenolic content obtained per gram of dried sugarcane bagasse in this study (5.28 mg GAE/g) was lower than the average of 7.83 mg GAE/g reported in other studies [11]. This difference could be related to the number of extractions. The TPC yield obtained in this study is higher than the results in other reports (1.78–4.26 mg GAE/g) [14].

Compared with molasses, the TPC extracted from sugarcane bagasse is higher than the 3.91 mg GAE/g obtained from B-type molasses, a by-product of the sugar industry [30]. It is also higher than the 3.75 mg GAE/g from molasses retrieved from sugar mills in Pakistan [31].

This study’s TPC from sugarcane bagasse is lower than the TPC extracted from *Citrus limetta* bagasse (20.38 mg GAE/g) [32]. It is also lower than the TPC extracted from six different white and red wine grape waste, valued within a range of 32 to 59 mg GAE/g [33]. The importance of the total phenolic content from sugarcane bagasse is based on the ease of acquiring the raw material.

### 3.3. Antioxidant Activity of SCB Extracts

The fresh sugarcane extract showed a percentage of DPPH radical inhibition of 71.32% (Table 2), which is similar to the 60% inhibition obtained from sugar cane bagasse in other studies [11]. A higher percentage of DPPH radical inhibition was obtained in the SCB than in sugarcane juice (42%) [34].

The Jujube fruit is known for its powerful antioxidant properties and essential nutritional contributions such as high vitamin C content, potassium, and fatty acids [35]. Compared to the mentioned fruit, the average (71.32%) of the rate of inhibition of DPPH radical from SCB (Table 2) is comparable to the 80% reported for Jujube extracts [35].

The ferric reducing power of 941.61 μmol/g from this study is similar to the 1011 μmol/g obtained from different jujube fruit samples in another study [35]. The 941.61 µM FeSO_4_ obtained in the fresh extract in this study (Table 2) is higher than the 132.35 µM/g FeSO_4_ from B-type molasses needed to inhibit the 50% of the free radicals [30].

### 3.4. Antimicrobial Activity of SCB Extracts

**Figure 1:**
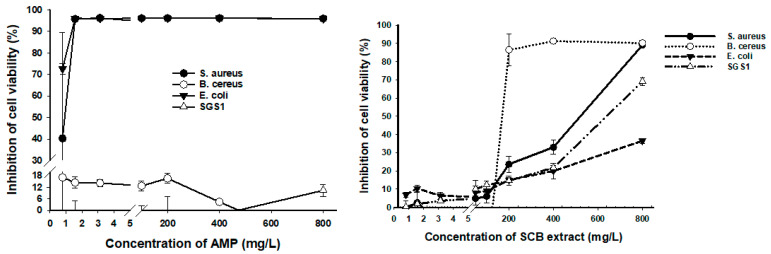
Effect of different concentrations of Ampicillin (AMP) and sugarcane bagasse (SCB) extracts on inhibition of cell viability of various foodborne pathogens and modified yeast. SGS1 = Slow Growth Suppressor 1, AMP = Ampicillin, SCB = Sugarcane bagasse, SGS1 = Slow Growth Suppressor 1. The curves show means ± SD (*n* = 3) for each concentration.

The DMSO control did not affect any of the microorganisms used for testing. The control sample containing DMSO showed no microbial growth, assuring the sterility of the dilutions. The effect of different concentrations of AMP and SCB extracts on inhibition of cell viability of various foodborne pathogens, and modified yeast is shown in Figure 1. The results indicate that SCB extracts had a small effect on *E. coli K12*, which is responsible for urinary, nervous, and enteric infections in its pathogenic form. 36.5% of cell viability inhibition was obtained at a concentration of 800 mg/L, while AMP had 72.56% inhibition at 0.78 mg/L against this gram-negative bacterium. SCB extracts also showed an effect on *S. aureus*, which is a gram-positive microorganism responsible for skin and bloodstream infections, at 800 mg/L with almost 90% inhibition. Still, AMP inhibited over 90% at a lower concentration of 1.56 mg/L. One bacteria responsible for food poisoning is the gram-positive *B. cereus*, and the antibiotic AMP showed no effect at the concentrations tested. However, SCB extracts had 86.49% average cell viability inhibition at a concentration of 200 mg/L. The modified yeast *SGS1* is related to the human homolog BLM and WRN responsible for Bloom syndrome and Werner syndrome. SCB extracts showed 69.14% and AMP 10.4% of inhibition at an equal concentration of 800 mg/L, respectively.

AMP shows a minimum inhibition at 0.8 mg/L against *S. aureus*, *B. cereus*, and *E.coli*, while the minimum inhibition for *SGS1* was above 400 mg/L (Figure 1). The minimum inhibition concentration (MIC) of SCB is 12.5 mg/L for *S. aureus*, 200 mg/L for *B. cereus*, 0.8 mg/L for *E.coli*, and 0.8 mg/L for *SGS1*. SCB showed a higher MIC than other phenolic extracts present in annatto seeds and leaves [15].

Our standardized extraction method of bioactive compounds from the SCB showed higher cell viability inhibition at lower concentrations than previous studies [15]. While the inhibition concentration of annatto seed extract for *B. cereus* was above 100 mg/L [15], SCB showed inhibition at concentrations of 12.5 mg/L [15] (Figure 1). SCB showed inhibition of *E. coli* and *SGS1* at concentrations as low as 0.8 mg/L. Our results agreed with previous findings, showing that SCB can change cell morphology and internal structure, provoking cell death [5]. The sugarcane bagasse extract showed higher inhibition on gram-positive bacteria than gram-negative bacteria (Figure 1). The difference can probably be explained by the different cell membrane structure between gram-positive and gram-negative bacteria [36].

### 3.5. Identification of Bioactive Compounds in Sugarcane Bagasse with HPLC-MS.

Table 3 shows the identified compounds in sugar cane bagasse extract. Madecassoside (Ms) is a terpene isolated from Centella Asiatica. This terpene has been reported to have anti-inflammatory and anti-depressant effects. In an in vivo study, it accelerated the recovery of burns in mice due to its antioxidant properties [37]. Sennoside B (SB), isolated from *Cassia pumila*, showed antimicrobial activity against *Rhizoctonia bataticola*, a pathogenic fungus that affects soybean and several crops [38]. Luteolin-7-O-glucoside (L7O) inhibited the growth of human colon adenocarcinoma cell lines, and in rats, it reduced the number of abnormal glands that lead to colon cancer untreated [39]. Isorhamnetin-3-O-rutinoside (I3OR) is the most abundant compound in sugarcane bagasse. I3OR inhibited 50% of the proliferation of human chronic myelogenous leukemia CML cell line K562 at 500 µg/mL [40].

(-)-Podophyllotoxin (Ptox) is a lignan that is an anticancer and a therapeutic agent against venereal warts. This secondary metabolite has minimal known natural sources [41].

The bioactive compounds found in this study are different than those identified in other SCB studies [14]. However, these compounds have in common the same pathway. Kaempferol, hesperidin, and isorhamnetin were identified, and they are metabolites of the compound naringenin. In previous studies, genistein, quercetin, and p-coumaric acid were identified in SCB [11]. Genistein and quercetin are metabolites of naringenin, while coumaric acid is a precursor of naringenin. In another study, 4-ethylphenol, 3-propylphenol, 2-methoxyphenyl, among others, were also identified in SCB [14]. 4-ethylphenol is a metabolite of p-coumaric acid. Therefore, although the bioactive compounds identified in these studies are different, they are related to the same chemical pathway.

### 3.6. Microencapsulation of Bioactive Compounds

Table 4 shows the effect of MD and Arabic GA on the encapsulation efficiencies and antioxidant activity of SCB extracts. The cubic model’s regression coefficients showed that MD, GA, and the interaction of both micro-encapsulating agents have a direct effect (*p* < 0.05) on the EE of freeze-dried SCB bioactive compounds. The highest (*p* < 0.05) EE of 83% was obtained using GA and no MD, and the lowest (*p* < 0.05) EE (40%) was obtained with MD and no GA. Other authors also found higher EE with increasing GA concentrations compared to MD [42].

The antioxidant activity of SCB extracts was higher (*p* < 0.05) when micro-encapsulated with MD or GA alone. Mixing MD and GA decreased (*p* < 0.05) the antioxidant activity of the micro-encapsulated bioactive compounds, measured with the FRAP method (Table 4). The lowest (*p* < 0.05) antioxidant activity was measured with an MD/GA ratio of 7.2/2.8 (%). When measured by DPPH, the combination of MD and GA did not affect (*p* > 0.05) the antioxidant activity of SCB.

GA has a higher molecular weight in comparison to MD, which can affect the pore size distribution and morphology of the freeze-dried powders, affecting EE and antioxidant activity (Table 4) [43]. MD has low emulsifying properties and surface-activity, which lowers its EE compared to GA [44].

The experimental design to micro-encapsulate the SCB bioactive compounds had a desirability index of 0.974. All the results from the optimal micro-encapsulated product (EE = 79.87%, FRAP = 105.02 µmol/gr, DPPH = 36.25%) were within the 95% predicted confidence interval, giving a final positive validation to the experimental design. This experiment’s optimal microencapsulation can be obtained with a ratio of 0.6% MD/9.423% GA. 

### 3.7. Shelf-Life Stability of SCB Extracts

The antioxidant activity of the sugarcane bagasse extract measured by DPPH, FRAP, and SOD assays was tested for any change after one month of storage at 4 °C. Table 2 shows the results from a paired multivariate t-test where each univariate test was significant. Therefore, the average of the differences in the percentage of inhibition of DPPH, the µM of FeSO_4_·7H_2_O equivalents (FRAP), and the rate of inhibition of SOD between the fresh and the stored extract was slightly different (*p* < 0.05).

The antioxidant capacity of SCB extracts maintained its stability after 30-day storage compared to the fresh extracts (Table 2). The stability obtained in this study is comparable with the results obtained from *Anemopsis californica* extracts, where the antioxidant capacity showed stability after 60 days of storage in the dark between 4 and −20 °C [45]. The same antioxidant stability was observed from *Piper betle* extracts after 180 days of storage in the darkness at 5 °C, retaining over 95% of the antioxidant capacity [46].

All results from antioxidant capacity tests in this study showed that the extracts from sugarcane bagasse could be stored at 4 °C for at least one month without losing its antioxidant capacity. These results provide useful information for industrial purposes because they offer a period in which the extracted bio compounds can be transformed into a food additive by microencapsulation.

### 3.8. Shelf-Life Stability of Free and Microencapsulated Bioactive Compounds

**Figure 2:**
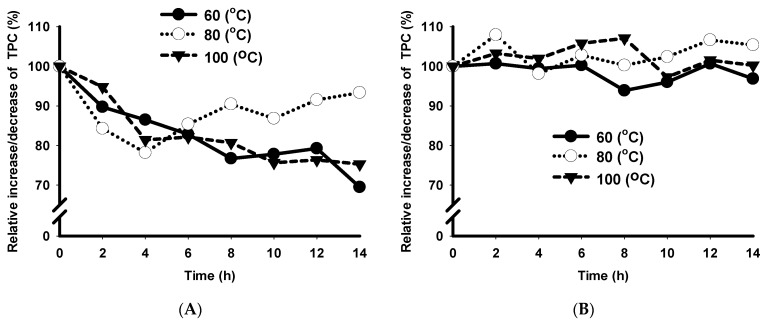
Effect of different storage time and temperatures on shelf-life stability of bioactive compounds from sugarcane bagasse. TPC = total phenolic content, (**A**) free compounds extract, (**B**) microencapsulated compounds extract. The curves show means ± SD (n = 3) for each concentration.

The storage temperature, time, and the interaction of both factors affected (*p* < 0.05) the TPC in the fresh extracts (Figure 2). Bioactive compounds were more stable (*p* < 0.05) at 80 °C than at 60 °C and 100 °C after 14 h of storage. The total phenolic content decreased by (*p* < 0.05) 31%, 7% and 25% after 14 h storage at 60 °C, 80 °C and 100 °C, respectively. TPC constantly decreased (*p* < 0.05) at 60 °C and 100 °C. After four hours, at 80 °C, TPC increased (*p* < 0.05), probably caused by reorganization and creation of new phenolic compounds [46].

Storage time and the combination of temperature and time affected the TPC of the microencapsulated extracts (Figure 2). After 14 h, microcapsules stored at 80 °C showed the highest TPC compared to 60 °C and 100 °C. Microcapsules held at 60 °C had a higher TPC than microcapsules stored at 100 °C (*p* < 0.05). At 80 °C, there was evidence of an upward trend due to increased bioactive compounds [46]. The overall temperature did not affect (*p* > 0.05) the TPC of the microencapsulated extracts. There is no difference in temperature between the TPC at time 0 and 14 h. There is no degradation of microencapsulated bioactive compounds caused by temperature over the timeframe used in this experiment. The results show that the TPC in the microencapsulated samples was more stable than the free extracts.

## 4. Conclusions

Sugarcane bagasse is an agro-industrial by-product with a high TPC. The results show that sugarcane bagasse extracts contain a high antioxidant activity of bioactive compounds. The antioxidant capacity of extracts remained stable for 30 days of storage at 4 °C, showing stability with no degradation, allowing further processing as a potential food additive or nutraceutical.

The bioactive compounds found in sugarcane bagasse have been reported as therapeutic and anticarcinogenic agents. The outcome of this research showed not only promising effects against well-known pathogenic bacteria but also as a possible anticancer agent. The microencapsulation of bioactive compounds by freeze-drying at the optimal parameters ensures the protection of the extracted bioactive compounds, positively supporting its potential use as a food additive. The optimal microencapsulation in this experiment can be obtained with a ratio of 0.6% MD/9.423% GA. Sugarcane bagasse is a good source of bioactive compounds. These compounds can be extracted from sugarcane bagasse, adding value to the sugarcane bagasse as a food additive or antimicrobial agent.

## Figures and Tables

**Table 1:** Forage analysis in sugarcane bagasse from Veracruz, Mexico.

	As Fed	Dry Matter
Moisture %	0.90 ± 0.07	---
Dry Matter %	99.10 ± 0.07	---
Crude Protein %	2.20 ± 0.35	2.20 ± 0.42
Available Protein %	1.40 ± 0.42	1.40 ± 0.42
ADICP %	0.80 ± 0.00	0.80 ± 0.00
Adjusted Crude Protein %	2.20 ± 0.35	2.20 ± 0.42
ADF %	55.30 ± 4.95	55.80 ± 4.95
aNDF %	81.90 ± 11.24	82.60 ± 11.31
Crude Fat %	0.50 ± 0.14	0.50 ± 0.14
TDN %	52.00 ± 0.71	52.00 ± 0.00
NEL, Mcal/Lb	0.19 ± 0.04	0.19 ± 0.04
NEM, Mcal/Lb	0.39 ± 0.02	0.40 ± 0.03
NEG, Mcal/Lb	0.15 ± 0.04	0.15 ± 0.04
Calcium %	0.07 ± 0.01	0.07 ± 0.00
Phosphorus %	0.04 ± 0.01	0.04 ± 0.01
Magnesium %	0.04 ± 0.03	0.04 ± 0.03
Potassium %	0.20 ± 0.06	0.21 ± 0.05
Sodium %	0.006 ± 0.00	0.006 ± 0.00
Iron ppm	1110.00 ± 63.64	1120.00 ± 77.78
Zinc ppm	9.00 ± 0.71	9.00 ± 1.41
Copper ppm	4.00 ± 0.71	4.00 ± 0.71
Manganese ppm	36.00 ± 1.41	36.00 ± 2.12
Molybdenum ppm	8.20 ± 0.85	8.30 ± 0.92
Sulfur %	0.04 ± 0.00	0.04 ± 0.00
Ash %	4.47 ± 0.71	4.51 ± 0.00
Soluble Protein % CP	---	42.00 ± 3.54
NFC %	10.00 ± 0.85	10.10 ± 0.85

ADICP = Acid Detergent Insoluble Crude Protein, ADF = Acid Detergent Fiber, aNDF = amylase and sodium sulfite treated Neutral Detergent Fiber, TDN = Total Digestible Nutrients, NEL = Net Energy Lactation, NEM = Net energy of maintenance, NEG = Net energy for gain, CP = Crude Protein, NFC = Non-Fiber Carbohydrates.

**Table 2:** Comparison of antioxidant capacity of sugarcane bagasse extracts before and after 30 days of storage at 4 °C.

Assay	Fresh Extract	Extract after 30 Days of Storage	Statistical Difference	T Statistic
DPPH (%)	71.32 ± 1.47 *	78.71 ± 1.16	−7.39 ± 2.04	−10.84
FRAP (µmol/g)	941.61 ± 7.03	1005.50 ± 10.53	−44.52 ± 30.82	−4.33
SOD (%)	27.10 ± 2.32	29.69 ± 3.04	−2.59 ± 3.71	−2.09

* SD = Standard deviation of the mean.

**Table 3:** Bioactive compounds found in a freeze-dried extract from sugarcane bagasse by HPLC-MS.

Biocompound	*m*/*z* Similarity	Mass (Da)	AA	RSD (%)	Classification
Isorhamnetin-3-O-rutinoside	979	623.42	651	0.2	Flavonoids
Madecassoside	999	973.39	469	5.4	Terpenes
Sennoside B	999	861.28	443	5.7	Anthranoids
Luteolin-7-O-glucoside (Cynaroside)	969	447.34	260	9.6	Glycosyloxyflavones
Kaempferol-3-Glucuronide	968	461.33	135	0.4	Flavonoids
Phosphatidylcholine 18	1000	842.65	134	0.4	Phosphatidylcholines
Hesperidin	983	609.41	119	1.6	Flavonoids
Thalsimidine	993	621.4	104	1.7	Alkaloids
Isorhamnetin-3-O-galactoside-6″-rhamnoside	987	623.36	93	0.5	Flavonoids
(-)-Podophyllotoxin	983	459.31	72	0.7	Lignans
Speciofiline (Uncarine F)	999	367.21	56	2.7	Alkaloids
Gardnerine	992	369.31	44	1.1	Alkaloids
Licoricesaponin G2	998	837.51	30	1.9	Saponiin
Pseudojervine	999	632.39	28	5	Alkaloids
Ginsenoside Rb1	994	1107.36	26	2.6	Saponin
Saikosaponin a	833	778.52	23	2.1	Saponin

AA = Absolute abundance (given as number of ions in the mass spectrometer), RSD = Relative standard deviation.

**Table 4:** Regression coefficients for prediction of encapsulation efficiency (EE) and antioxidant activity of maltodextrin and gum arabic by freeze-drying of SCB bioactive compounds.

	EE (%)		FRAP (µmol/gr)		DPPH Inhibition (%)	
	Coefficient	*p*-Value *	Coefficient	*p*-Value *	Coefficient	*p*-Value *
X1	43.73	<0.0001	104.9	0.0004	30.24	0.0003
X2	83.11	<0.0001	107.53	0.0029	35.52	0.0002
X1 X2	48.22	<0.0001	−39.95	0.0101	−6.36	0.2736
X1X2(X1-X2)	49.30	0.0057	−111.02	0.0006	−38.13	0.0032
SD	2.83		4.56		1.98	
R2	0.9644		0.8274		0.8331	

* Significant at α = 0.05 level; X1 = Maltodextrin, X2 = Gum arabic.

## Data Availability

Not applicable.
